# Shock

**Published:** 1889-10-19

**Authors:** 


### SURGERY.

### SHOCK.

"Some Thoughts on Surgical Shock, with Special Reference to Brain and Spinal Operations" is the heading of an article by Dr. F. X. Dercum, neurologist to the Philadelphia Hospital.* Operations on the cerebrum, cerebellum, and cord are attended by profound shock, which often causes death. Everything possible should therefore be done to prevent shock. The first requirement is a condition of rest for several days before the operation. Emotional friends and relatives should be excluded. There should be a cheerful previous acquaintance with the surgeon and with a properly trained nurse. In addition, Dr. Dercum suggests the hypodermatic injection of strychnine just before the operation, which is indicated to prevent the general paralysis of the nervous system characterising shock. Whatever may be thought of this last suggestion, the first are worthy the attention of the surgeon in all cases where time and opportunity for carrying them out are allowed.

* *Philadelphia Medical News*, Sept. 21, 1889. † *Lancet*, Sept.

